# Utilization of Artificial Intelligence to Improve Equitable Healthcare Access for Breast Implant Patients

**DOI:** 10.1093/asjof/ojae093

**Published:** 2024-10-22

**Authors:** Louisa B Ragsdale, Aurora M Kareh, Rohun Gupta, Peter K Firouzbakht, Christina M Plikaitis, Katherine A Rodby

## Abstract

Recently, mandated FDA patient decision checklists were developed with the goal of improving the informed decision-making process for patients considering breast implants. However, these checklists are written at reading levels far higher than recommended by the National Institutes of Health and the American Medical Association. This study aims to improve the accessibility, and therefore, the utility of the mandated FDA patient literature for the average breast implant patient using the assistance of artificial intelligence (AI). Patient decision checklists were obtained from the 3 most utilized breast implant manufacturers in the United States—Allergan, Mentor, and Sientra. A novel patient decision checklist was synthesized by AI, written at the sixth grade reading level, using these checklists as source material. The AI-assisted checklist was edited by plastic surgeons for both formatting and content. The overall readability of Allergan, Mentor, and Sientra patient checklists correlated with the college reading level. These documents were of a statistically significantly higher reading level than the AI-assisted checklist, which was written at the recommended sixth grade level. Text composition analysis similarly demonstrated substantial differences between the AI-assisted and FDA-mandated literature. The currently mandated breast implant patient checklists are written at a college reading level and are inaccessible to the average patient. The authors propose a new patient decision checklist, generated with the assistance of AI, to improve healthcare access within plastic surgery. This simplified material can be used as an adjunct to the current checklists to improve shared decision making.

The National Institutes of Health (NIH) and the American Medical Association (AMA) recommend that all patient education materials should be written at or below the sixth grade reading level in order to ensure equitable healthcare access for the average patient.^1^ Despite these recommendations, patient literature across all sectors of medicine is consistently written at the college reading level.^2-9^ Plastic surgery patient literature is no exception to this precedent, with numerous studies confirming that these resources are written at reading levels that are inaccessible to the average patient.^7,9-12^

Given growing concerns regarding the safety of breast implants, the FDA recently mandated decision-making checklists and updated labeling, with the aim of improving the informed decision-making process for patients considering breast implantation.^13^ Yet, previous analysis of the FDA-mandated checklists by our group revealed that these documents are written at a college reading level, thereby limiting their utility for the average breast implant patient.^11^ Although the intention of these checklists is rooted in enhancing patient–physician communication, the mandatory nature of these documents constricts the breast implant patient to sign a form of which she may have little to no comprehension, therefore undermining the informed consent process.

Recent advances in the realm of artificial intelligence (AI) have given rise to large language model (LLM) chatbots, which use deep learning algorithms to both understand and generate human-like content. The most popular LLM, ChatGPT (Chat Generative Pre-Trained Transformer), was released to the general public in 2022 by OpenAI (San Francisco, CA) and has been applied in a seemingly limitless number of ways ever since. Following its release, plastic surgery has seen an exponential rise in publications regarding the application of ChatGPT to patient documentation, research, grant writing, and even the In Service Training Examination.^14-18^ Promisingly, ChatGPT has also been used to improve patient education materials within the realms of otolaryngology, ophthalmology, urology, and plastic surgery.^19-22^

This study seeks to improve the accessibility of the mandated breast implant literature by using AI to assist in synthesis of new breast implant patient materials, written at the NIH- and AMA-recommended reading levels. By creating breast implant patient education materials that are accessible to the average breast implant patient, we aim to mitigate the health disparity currently present within the field of plastic surgery and improve the shared decision-making process between surgeon and patient.

## METHODS

FDA-mandated breast implant patient education materials, including patient decision checklists and breast implant boxed warnings, were obtained from Allergan (Irvine, CA), Mentor (Irvine, CA), and Sientra (Franklin, WI), which are the most commonly used breast implant brands in the United States. All documents obtained were written in English. The educational materials were then input into ChatGPT-4, which is an open-source language processing tool driven by AI. Following, the AI platform was prompted to “create a new checklist, based on the input source material, which is written in checklist format and written at a sixth grade reading level.” This query was performed in August 2024. The synthesized checklist was subsequently edited for both content and formatting by the authors, with care taken to ensure all critical aspects of the FDA-mandated checklists were included (Appendix).

Readability and text analysis of both the mandated and AI-assisted checklists was performed using Microsoft Word (Version 2007, Microsoft Corporation, Redmond, WA) and 3 publicly available, online readability calculators (Readability Test, WebFX, Harrisburg, PA; Readability Formulas; Online Utility, Readability Calculator). The FDA-mandated checklists and the AI-assisted checklist were analyzed to determine Flesch Reading-Ease Score (FRES), Flesch–Kincaid Grade Level (FKGL), Gunning Fog (GFI) Index, Coleman–Liau Index (CLI), Simplified Measure of Gobbledygook (SMOG), and Automated Readability Index (ARI).

FRES is a scale of 0 to 100, with a score of 100 indicating language easily understood by the average 11 year old, whereas a score of 50 or less indicates language at a college reading level. FKGL, GFI, CLI, SMOG, and ARI are all proxies for grade level or years of education needed for comprehension. Scores >12 indicate the material is of college reading level. Further textual analysis included sentence count, word count, number and percentage of complex words, average number of words per sentence, and average number of syllables per word.

As FKGL, GFI, CLI, SMOG, and ARI are all surrogates for years of education, these scores were averaged into a composite score. Two-tailed 1-sample *t*-tests were performed to compare mean readability scores. *T*-tests were only performed on groups with 30 data points or more to ensure adequate power; thus, FRESs were not compared between groups, nor were aspects of text composition, such as word or sentence counts. *P*-values <.05 were considered to be significant. Data analysis was performed using IBM SPSS Statistics, Version 27 (IBM Corp., Armonk, NY).

## RESULTS

**Figure 1.**
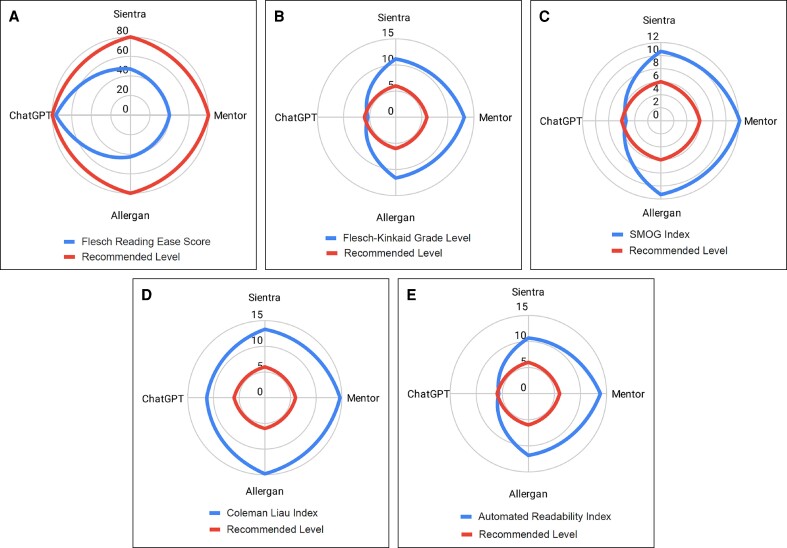
(A-E) Readability of Mentor, Sientra, Allergan, and ChatGPT-assisted checklists.

A total of 16 publicly available patient documents from Allergan, Mentor, and Sientra were included for analysis. The average readability of the FDA-mandated breast implant literature correlated with a college reading level (FRES: 43.2 ± 3.61, grade level: 13.1 ± 1.64). Conversely, readability analysis of the ChatGPT-assisted checklist correlated with a high sixth grade reading level (FRES 76.6, grade level 6.90). Mentor documents were the most difficult to read based on average FRES (39.7 ± 1.5) and grade level indices (13.8 ± 0.7). Sientra documents were found to have the easiest readability but were still at the college reading level (FRES: 47.1 ± 4.8, grade level: 11.8 ± 1.8; Figure 1, Table 1). Comparison of average Sientra, Mentor, and Allergan grade level indices to that of the ChatGPT-assisted checklist using 1-sample 2-tailed *t*-testing revealed the FDA-mandated documents to be written at significantly higher grade level than that of the AI-assisted checklist (*P* < .0001). Average FRES was not compared because of low sample size.

**Table 1.** Readability Analysis

	Flesch reading-ease		Flesch–Kinkaid grade level		Gunning fog score		SMOG index		Coleman liau index		Automated readability index		Composite grade level		
	Out of 100	SD	US grade level	SD	US grade level	SD	US grade level	SD	US grade level	SD	US grade level	SD	US grade level	SD	*P*-value
Sientra	47.1	4.8	11.1	1.6	13.2	2.6	10.6	1.4	13.4	0.6	10.7	2.5	13.07	1.65	<0.001
Mentor	39.7	1.5	13.1	0.6	15.4	1.3	12.2	0.7	14.7	0.2	13.8	0.8			
Allergan	42.8	2	11.6	0.9	13.9	1.4	11.3	0.8	14.9	0.7	11.8	1.2			
ChatGPT assisted	74.8	—	5.0	—	6.6	—	5.8	—	11.5	—	4.8	—	6.74	—	—

SD, standard deviation.

**Figure 2.**
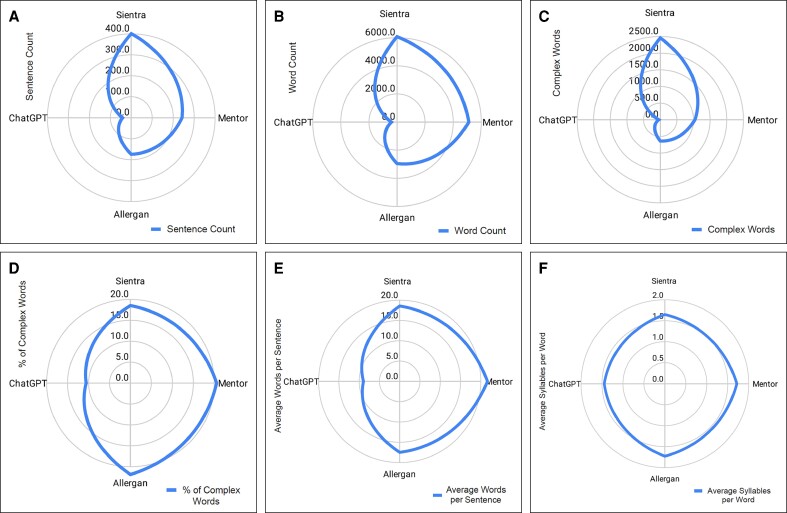
(A-F) Textual analysis of Mentor, Sientra, Allergan, and ChatGPT-assisted checklists.

Analysis of the FDA-mandated documents and the ChatGPT-assisted checklist similarly revealed differences in overall text composition. Average sentence count, word count, complex word count and percentage, average number of words per sentence, and average number of syllables per word were all substantially higher in the FDA-mandated group relative to the ChatGPT-assisted checklist. *T*-test analysis was not performed due to sample size (Figure 2).

## DISCUSSION

Across all sectors of medicine, patient literature is consistently written at reading levels far in excess of those as recommended by the AMA and NIH.^2-9^ The authors of this study have previously published on the readability of the FDA-mandated breast implant labeling, which is written at collegiate levels and is therefore of limited utility to the average breast implant patient.^11^ Although demonstration of this health disparity has inherent benefit, the authors wished to propose an actionable solution to this problem by creating a new breast implant checklist, written at the recommended reading sixth grade level.

**Figure 3.**
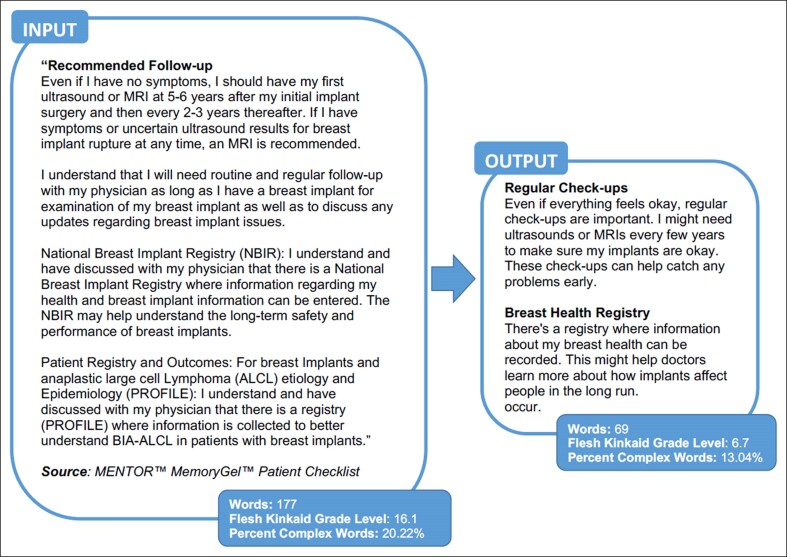
Demonstration of distillation of breast implant checklist content by artificial intelligence.

Recently, plastic surgery has seen a rapid expansion in the use of ChatGPT and other LLM AI programs, such as Gemini (formerly Bard) by Google (Google; Mountain View, CA), LLaMA by Meta (Meta; Menlo Park, CA), and most recently, Grok by xAI (xAI; San Francisco, CA). LLMs use machine learning to analyze large amounts of written data, from which they learn the structure and pattern of language. When prompted, this technology can effectively summarize lengthy texts, distilling it into a more palatable form, while still retaining its core content (Figure 3). Given these capabilities, LLM AI offers a potential solution to the issue currently posed by the FDA-mandated breast implant labeling and other college reading level patient literature.

In the modern era, medicine has shifted away from a paternalistic style towards a shared decision-making model, in which the patient and provider work together to formulate the best healthcare plan for the patient. Patient education materials empower patients within the shared decision-making model by promoting patient knowledge, and therefore, a patient's ability to participate in his or her care. However, the consistently high reading levels found in plastic surgery patient resources,^7,9,12,23^ including the newly mandated FDA breast implant labeling,^11^ perpetuate a style of medicine where patients are heavily reliant on physicians for comprehension, undermining the very essence of the shared decision-making process. American physicians have a literacy rate which is >95% of the general population,^24^ which in turn may account for physicians’ gross overestimation of their patients degree of literacy and extent of education.^25^ Although there is no doubt that most physicians expertly explain the mandated documents to their patients, patient materials written at the collegiate level foster dependence of the patient upon her physician's ability to distill this content into a palatable form, undermining each patient's agency within this process.

The ramifications of low health literacy for patients undergoing breast implantation are significant. Generally, low health literacy has been associated with increased emergency care and hospitalizations, poor adherence to medication regimen, and higher mortality rates.^26^ Breast cancer patients with low health literacy have been shown to have a significantly higher amount of unmet information needs^27^ and are less likely to undergo any breast reconstruction following mastectomy.^28^ In light of this data, simplified, and therefore more accessible, breast implant labeling has the potential to improve the health literacy of patients undergoing breast implantation. A promising study within the obstetrics and gynecology literature demonstrated that patients who were provided with a low health literacy version of a sterilization consent form had a significantly improved understanding of tubal ligation, regardless of their existing literacy level.^29^ The authors of this study believe the AI-assisted sixth grade reading level forms could have comparable effect within the breast implant population, potentially improving long-term outcomes as a result.

The FDA-mandated new breast implant labeling in 2021 with the goal of improving the informed decision-making process for patients considering breast implantation and better communicating the risks of these devices to patients.^13^ In light of our previous findings,^11^ we have petitioned the FDA to mandate these documents be rewritten at appropriate reading levels by their respective manufacturers. Because of the considerable amount of time required to enact change at the highest level, our practice elected to supplement the cumbersome FDA-mandated materials with documents that more evenly match the health literacy of our patients. By successfully employing AI assistance to rewrite the FDA patient checklists at the recommended sixth grade reading levels, we aim to significantly better align these materials with the initial intention of the FDA, which in turn increases health literacy and improves the utility of this checklist for all breast implant patients.

The authors of this study are the first to acknowledge that the new AI-assisted checklist cannot replace the legally mandated breast implant labeling. Rather, we hope that this concise and easy to read document can be used as an adjunct to decrease the burden posed on physicians by the current literature and to improve physician–patient communication. In our practice, patients are provided a copy of the AI-assisted checklist when first roomed in the clinic, allowing the patient to review this brief checklist while waiting for her provider. As this checklist contains all of the salient points of the FDA-mandated checklist, we find that patients are empowered to ask more questions during their appointments and generally have a better understanding of breast implantation following. We believe using the AI-assisted checklist in this adjunctive manner significantly improves the experience for both the patient and her physician.

There is no doubt that medicine is on the cusp of an AI revolution as the applications of this technology are seemingly limitless. Nevertheless, the authors acknowledge that LLMs are still in their relative infancy and have heeded the warnings published regarding blind-faith use of this technology.^22,30,31^ In light of the above, the authors decided to use AI to assist with the creation of a new checklist, rather than synthesize 1 in its entirety. When used in this way, we believe AI to be a powerful adjunct to the mind of the physician, capable of improving the patient experience, and ultimately, healthcare outcomes.

## CONCLUSIONS

The currently mandated breast implant patient checklists are written at a college reading level and are inaccessible to the average patient. We propose a new patient decision checklist, generated with the assistance of AI, in order to improve healthcare access within plastic surgery. This simplified material can be used as an adjunct to the current checklists in order to improve shared decision making.

## Supplemental Material

This article contains supplemental material located online at https://doi.org/10.1093/asjof/ojae093.

## Supplementary Material

ojae093_Supplementary_Data
