# Supplementary material for: Utilization of Artificial Intelligence to Improve Equitable Healthcare Access for Breast Implant Patients
Source: Aesthet Surg J Open Forum. 2024 Oct 22;6:ojae093. doi: 10.1093/asjof/ojae093 (PMC11646116; doi:10.1093/asjof/ojae093)
Supplement: ojae093_Supplementary_Data [file ojae093_supplementary_data.pdf]

Breast Implant Consent Form Summary

Thinking About Breast Implants?

This sheet will help you know the important things to think about before surgery. Talk to your doctor and ask questions.

Who Should NOT Get Breast Implants

- Do I have an infection right now?
- Do I have breast cancer that hasn't been treated?
- Am I pregnant or breastfeeding?

If you answered "yes," breast implants might not be safe for you.

Who Might Have More Risks

- Do I have a health problem that makes healing harder?
- Am I a smoker, or did I used to smoke?
- Am I taking meds that weaken my immune system?
- Will I need radiation or chemo?
- Do I have blood problems?
- Do I have bad blood flow in my breasts?

If you answered "yes," talk to your doctor. You might have more risks with breast implants.

Things That Aren't Fully Known

- Do I or my family have immune issues?
- Do I have mental health issues?
- Do I have something else already inside my breasts?

These things are not fully studied, so ask your doctor for more information.

Risks of Breast Implant Surgery

- Breast pain
- Changes in how your skin or nipples feel
- Breasts not looking the same
- Infection
- Scars
- Swelling
- Bleeding
- Fluid around the implant
- Tight scar tissue around the implant
- Trouble breastfeeding
- Needing more surgeries later

Talk to your doctor about these risks.

Breast Implant Related Cancer (BIA-ALCL)

- Implants can be linked to a cancer called BIA-ALCL.
- It happens more with textured implants but can happen with smooth ones.
- Watch for swelling, tightness, pain, lumps, or swelling years after getting implants.
- Treatment might mean removing the implants and the tissue around them. Some people may need chemo or radiation.

Ask your doctor to explain more about this cancer.

Breast Implant Illness

- Some people with implants feel tired, have joint pain, rashes, or memory problems.
- Some feel better after removing implants, but not all.
- We need more research to know why this happens.

If you feel like this, talk to your doctor.

Long-Term Risks

- Implants do not last forever.
- You may need more surgeries later.
- Implants can break, leak, or move.
- Silicone from implants can leak into your body.
- Implants can make it hard to find breast cancer with a mammogram.

Your doctor will tell you how to care for your implants over time.

Follow-Up Care

- If you have silicone implants, you'll need imaging tests 5-6 years after surgery, then every 2-3 years.
- If you notice any issues, call your doctor right away.
- Go to regular check-ups with your doctor.

Know Your Options

- You can choose not to have reconstruction after a mastectomy.
- You can use your own tissue (autologous reconstruction) or breast implants.
- Breast augmentation (to make breasts bigger) is optional but has risks.
- Removing implants later may cause dimples or sagging.

Talk to your doctor about all your options.

Confirm

- I have read and understood the information.
- I have talked to my doctor about the risks and benefits of breast implants.
- I have asked all my questions, and my doctor has answered them.
